# Latin-American challenges and opportunities in rheumatology

**DOI:** 10.1186/s13075-017-1247-7

**Published:** 2017-02-10

**Authors:** Francisco Airton Castro Rocha

**Affiliations:** Department of Internal Medicine, Federal University of Ceará, Rua Coronel Nunes de Melo, 1315, CEP: 60430-270, Rodolfo Teofilo, Fortaleza, CE Brasil

**Keywords:** Rheumatology, Latin America, Health

## Abstract

The importance of diseases to mankind is very similar worldwide. However, social and environmental issues, not to mention political and individual aspects, affect the prevalence and management of diseases and their outcomes. I present a brief and tentative comment to illustrate that the Latin-American rheumatology community, despite relevant gains in recent years, has some challenges to face in order to improve patient care in our region.

Recent years have seen great achievements in the rheumatology field with improvements in disease management, particularly of rheumatoid arthritis (RA). Challenges faced by mankind considered as urgencies in some areas of the globe may be seen otherwise in other regions. Medical issues are no exception. Considering that priorities vary within specific countries, it is difficult to evaluate challenges and demands affecting the Latin-American “subcontinent”. The present comment should be considered in the context of these limitations, given that an area comprising 19 countries and the Caribbean region, with circa 600 million inhabitants, has issues pertaining to specific countries and relevant variations within countries may apply [1].

## Manpower requirements

The impact of increased life expectancy, ageing populations, and obesity on the epidemiological importance of musculoskeletal diseases worldwide justifies an increase in manpower. The number of rheumatologists in Latin-America (LA) is considered insufficient compared to developed countries. In addition, their unequal distribution worsens the deficit. The vast majority of professionals work in urban areas of major cities, leaving a large number of individuals without access to rheumatologists [1, 2]. Data from 2012 showed a mean ratio of 157,809 inhabitants per rheumatologist among 1229 rheumatologists practicing in Brazil. Analyzing the 27 Brazilian States and the Federal District, ratios varied from 41,383 to 758,786 inhabitants per rheumatologist, displaying a positive correlation with the local gross domestic product and indices of human development [2]. This uneven distribution in Brazil mimics the situation in LA; variation from as low as 0.11 up to 4.83 rheumatologists per 100,000 inhabitants in Nicaragua and Uruguay, respectively—with the rheumatologists working predominantly in capitals and large cities—has been reported [1].

The distribution of pediatric rheumatologists is even more unequal, with 94% working in only 6 out of 19 countries in LA (Argentina, Brazil, Chile, Colombia, Cuba, and Mexico), with these confined mainly to large cities [1, 2].

We should remark that the number of rheumatologists increased in all countries in LA but Chile between 2012 and 2015, with an estimated 4603 specialists in 2015. This was associated with an increase in the number of training programs [1] and we may speculate that recent improvements in the management of autoimmune diseases attracted young physicians to the specialty.

With regard to health care professionals, including nurses, nutritionists, physiotherapists, and psychologists, which are all partners in the multidisciplinary team to better treat rheumatologic patients, training to provide specialized care rather than increasing their numbers may be the most urgent need [3].

## Medications and health facilities

The unequal manpower distribution is mirrored by access to medications and health care facilities. Low income as well as lower literacy have been associated with delayed and irregular use of biologic disease-modifying antirheumatic drugs (DMARDS), thereby affecting disease outcome [4]. The social burden of the uneven economic distribution in LA is also reflected in access to health care, meaning better opportunities for those with high incomes living in developed areas.

There are claims that the coverage of health systems varies from 22% in Paraguay to 100% of the population in Argentina, Brazil, and Cuba [5]. However, a lack of individual resources to seek institutions, cultural concepts assuming that “rheumatism” is a mild problem and that rheumatic diseases are “irreversible and incurable” and caused by the natural ageing process, late referral by primary care physicians, and a lack of knowledge about a referral center are some reasons to explain the delay in initiating RA treatment in Brazil [4]. Despite limitations, RA treatment has greatly improved, diagnosis is easier, and access to non-biologic DMARDS is relatively universal in LA. On the other hand, delays in diagnosing spondyloarthropathies and other less prevalent diseases, including systemic lupus erythematosus and vasculitis, continues to impose a burden that is undocumented in LA.

Data regarding access to biologic DMARDS are inconsistent, which is coupled to large differences among countries. In Brazil, the public health system provides free access to any patient with an RA diagnosis that failed non-biologic DMARDS; these patients are usually treated in public institutions linked to tertiary rheumatologic training services. This represents over 50% of the Ministry of Health’s budget for free medications for chronic diseases [6, 7]. However, a survey conducted among 212 rheumatologists working in LA showed that biologic DMARDS are guaranteed to less than 10% of RA patients, with a major negative socioeconomic impact [5]. The emergence of biosimilars, proposed to lower costs, came with worries about regulation of their use given the complex characteristics of the compounds and the availability of the so-called “intended copies” of biologic DMARDS. Regulatory issues are still being discussed and they obviously differ among countries. Rheumatologists and patient groups should thus work with health authorities in order to cut prices and increase access without compromising quality [7, 8].

Challenges regarding non-pharmacological treatments may have been overlooked. Rehabilitation programs and facilities, nutrition support, and psychological treatment are far from ideally offered in LA. Recommendations for osteoarthritis (OA) management consider non-pharmacological strategies as at least as important as medications in providing pain relief and retarding disease progression. It has even been suggested that non-pharmacological approaches could prevent knee OA [9]. Thus, increased health facility capacity, initiatives for patient education, and strategies for weight control—providing benefits to a high number of individuals at relatively low cost—are at least as urgently needed in LA as access to high cost biologic therapies. In keeping with this assumption, patient-reported outcomes (PROs) are increasingly relevant to assess the quality and effectiveness of management strategies and to facilitate decisions about whether or not they should be incorporated in health care [10]. Health systems in LA may thus improve assistance and optimize costs using PRO experience.

## Research

Collecting specific data on epidemiology across LA is probably our most urgent need. A recent report on the “global” burden of hip and knee OA included almost no data from LA [11] despite the World Health Organization–International League of Associations for Rheumatology COPCORD (Community-Oriented Program for Control of Rheumatic Disease) having shown, as expected, that OA is the most prevalent rheumatic disease in LA [1]. Facing opportunities, recent abstracts of the Pan-American League of Associations of Rheumatology (PANLAR) meeting emphasized specificities of cohorts and registry data from LA [12]. The recent outbreak of arbovirus infections in LA, particularly joint involvement in Chikungunya fever [13], is another example demonstrating the varying interests in across the globe.

## Conclusions

The efforts of rheumatologists working in LA to tackle their own problems have yielded improvements and we hope this will be an ever-growing process, resulting in advances in rheumatology care.

## Abbreviations

COPCORD: Community-Oriented Program for Control of Rheumatic Disease
DMARD: Disease-modifying antirheumatic drug
LA: Latin America
OA: Osteoarthritis
PANLAR: Pan-American League of Associations of Rheumatology
PRO: Patient reported outcomes
RA: Rheumatoid arthritis.
